# Lineage tracing for multiple lung cancer by spatiotemporal heterogeneity using a multi-omics analysis method integrating genomic, transcriptomic, and immune-related features

**DOI:** 10.3389/fonc.2023.1237308

**Published:** 2023-09-14

**Authors:** Yijun Song, Jiebai Zhou, Xiaotian Zhao, Yong Zhang, Xiaobo Xu, Donghui Zhang, Jiaohui Pang, Hairong Bao, Yuan Ji, Mengna Zhan, Yulin Wang, Qiuxiang Ou, Jie Hu

**Affiliations:** ^1^ Department of Pulmonary and Critical Care Medicine, Zhongshan Hospital, Fudan University, Shanghai, China; ^2^ Geneseeq Research Institute, Nanjing Geneseeq Technology Inc., Nanjing, China; ^3^ Department of Pulmonary and Critical Care Medicine, Shanghai Geriatric Center, Shanghai, China; ^4^ Department of Pathology, Zhongshan Hospital, Fudan University, Shanghai, China

**Keywords:** lineage tracing, multiple lung cancer, spatiotemporal heterogeneity, multi-omics analysis method, multiple primary lung cancer (MPLC), intrapulmonary metastasis (IPM)

## Abstract

**Introduction:**

The distinction between multiple primary lung cancer (MPLC) and intrapulmonary metastasis (IPM) holds clinical significance in staging, therapeutic intervention, and prognosis assessment for multiple lung cancer. Lineage tracing by clinicopathologic features alone remains a clinical challenge; thus, we aimed to develop a multi-omics analysis method delineating spatiotemporal heterogeneity based on tumor genomic profiling.

**Methods:**

Between 2012 and 2022, 11 specimens were collected from two patients diagnosed with multiple lung cancer (LU1 and LU2) with synchronous/metachronous tumors. A novel multi-omics analysis method based on whole-exome sequencing, transcriptome sequencing (RNA-Seq), and tumor neoantigen prediction was developed to define the lineage. Traditional clinicopathologic reviews and an imaging-based algorithm were performed to verify the results.

**Results:**

Seven tissue biopsies were collected from LU1. The multi-omics analysis method demonstrated that three synchronous tumors observed in 2018 (LU1B/C/D) had strong molecular heterogeneity, various RNA expression and immune microenvironment characteristics, and unique neoantigens. These results suggested that LU1B, LU1C, and LU1D were MPLC, consistent with traditional lineage tracing approaches. The high mutational landscape similarity score (75.1%), similar RNA expression features, and considerable shared neoantigens (n = 241) revealed the IPM relationship between LU1F and LU1G which were two samples detected simultaneously in 2021. Although the multi-omics analysis method aligned with the imaging-based algorithm, pathology and clinicopathologic approaches suggested MPLC owing to different histological types of LU1F/G. Moreover, controversial lineage or misclassification of LU2’s synchronous/metachronous samples (LU2B/D and LU2C/E) traced by traditional approaches might be corrected by the multi-omics analysis method. Spatiotemporal heterogeneity profiled by the multi-omics analysis method suggested that LU2D possibly had the same lineage as LU2B (similarity score, 12.9%; shared neoantigens, n = 71); gefitinib treatment and *EGFR*, *TP53*, and *RB1* mutations suggested the possibility that LU2E might result from histology transformation of LU2C despite the lack of LU2C biopsy and its histology. By contrast, histological interpretation was indeterminate for LU2D, and LU2E was defined as a primary or progression lesion of LU2C by histological, clinicopathologic, or imaging-based approaches.

**Conclusion:**

This novel multi-omics analysis method improves the accuracy of lineage tracing by tracking the spatiotemporal heterogeneity of serial samples. Further validation is required for its clinical application in accurate diagnosis, disease management, and improving prognosis.

## 1 Introduction

Up to 20% of patients in screening populations are diagnosed with multiple lung cancer with synchronous or metachronous lung tumors, compared to a previous estimation that only 0.8%–4% of patients had multiple lesions (1). The identification of multiple pulmonary nodules raises a crucial clinical issue of whether multiple pulmonary nodules represent intrapulmonary metastasis (IPM) with a common origin or multiple primary lung cancer (MPLC) with independent lineages. Chest computed tomography (CT) for high-risk individuals has been endorsed with the increased awareness of clinicians regarding MPLC screening (2). MPLC is more likely to develop in the upper lobes of both lungs, with approximately 50%–70% of patients with multiple lesions having the same pathological types (3). Given that MPLC patients have better prognoses than IPM patients, distinguishing between MPLC and IPM holds clinical significance in staging, prognosis prediction, and therapeutic intervention development (4). For instance, MPLC patients are generally treated with aggressive curative therapy, whereas palliative therapy is typically employed for IPM cases (5). However, differentiating between MPLC and IPM remains a significant challenge, particularly for common histological subtypes and lesions undergoing radiotherapy, leading to ambiguous diagnoses and patient management (6).

Pathological evaluation plays a vital role in the determination of MPLC versus IPM (7). The first clinicopathologic criteria proposed by Martini and Melamed (MM criteria) in 1975 have been proven to be insufficient for distinguishing MPLC (8–10) owing to only taking lung cancer histological types into consideration and the overlap of major histological subtypes. As a result, the International Association for the Study of Lung Cancer proposed a staging category, in which pathologic criteria based on comprehensive histological assessment were supplemented by radiographic appearance, rates of growth, and biomarkers (6, 7, 11). In addition, the eighth edition American Joint Committee on Cancer staging manual incorporated clinical, histopathological, and molecular diagnoses (12). Furthermore, a novel image-based algorithm was developed to identify IPM by imaging and clinical features. For example, solid lesions without spiculation or air bronchogram suggest IPM (13). Nevertheless, both comprehensive histological assessment and imaging algorithms have limitations resulting from inter-observer variability and the expertise of specialists, leading to indeterminate classifications and misclassifications.

Integrating multiple molecular features provides a powerful approach for lineage tracing leveraging spatiotemporal heterogeneity. Mutations in proto-oncogenes, such as *EGFR*, *KRAS*, *BRAF*, and *ALK*, can be used as molecular markers with a concordance of 70% between morphologic and molecular classifications (9). However, relying solely on common driver mutations as the lineage indicators might raise issues due to their relatively high prevalence (14). Array comparative genomic hybridization has been used to define recurrences in MPLC (15), and the implementation of next-generation sequencing (NGS) enables more comprehensive profiling from genome to transcriptome, thereby enhancing the accuracy of lineage tracing by molecular features (16). For instance, Murphy et al. conducted a diagnostic lineage test based on genomic rearrangements through mate‐pair sequencing in which the unique signatures of somatic junctions presented ideal markers to distinguish MPLC (16, 17). The partnering of molecular testing and clinicopathologic data can also increase the accuracy of lineage tracing (4, 18). To enhance the reliability of distinguishing MPLC from IPM, a multidisciplinary approach is required to overcome the limitations of using a single criterion-based approach.

In this study, we aimed to develop a multi-omics analysis method, integrating whole-exome sequencing (WES) data, transcriptome sequencing (RNA-Seq) data, and neoantigen prediction, to improve lineage tracing accuracy. We defined the lineage of tumors collected at multiple time points from two patients with multiple lung cancer using genetic mutational profiles, RNA expression levels, and tumor neoantigens. Furthermore, we compared the results of this novel multi-omics analysis method with those of conventional clinicopathologic approaches and a novel imaging-based approach.

## 2 Materials and methods

### 2.1 Patient inclusion

A male lung cancer patient (LU1) diagnosed with adenocarcinoma in November 2015 was enrolled. After initial diagnosis, six tumor tissue samples were serially collected between 2015 and 2021, including two adenocarcinoma samples, two minimally invasive adenocarcinoma (MIA) samples, one small cell lung cancer (SCLC) sample, and one large cell neuroendocrine carcinoma (LCNEC) sample. Additionally, one potential tumor biopsy, which was ultimately identified as a benign sample, was also collected. Another female lung cancer patient (LU2) diagnosed with adenocarcinoma in January 2012 was enrolled. Between 2012 and 2021, four tumor tissue samples were collected, including three adenocarcinomas and one SCLC sample. A total of 11 formalin-fixed and paraffin-embedded (FFPE) tissue samples were subject to WES and RNA-Seq. The study was approved by the Ethics Committee of Zhongshan Hospital Fudan University (approval number: B2017-142R) and in accordance with international standards of good clinical practice. Two patients provided signed written informed consent to participate in this study.

### 2.2 Lineage tracing by multiple approaches

The multi-omics analysis method integrated the genomic, transcriptomic, and tumor neoantigen features based on WES and RNA-Seq data. Two experienced pathologists blindly performed independent histological reviews on tumor samples according to the WHO classification (8). Based on histological criteria suggested by Girard and Detterbeck et al., samples were classified as primary if either of the paired nodules was adenocarcinoma *in situ* or MIA or if the predominant histopathologic pattern was different between paired nodules. Samples with similar histological appearance to a primary cancer were classified as metastasis if they were not judged to be primary or multiple foci of lepidic predominant adenocarcinoma, adenocarcinoma *in situ*, or MIA (6, 7). In addition to the pathology method, lineage tracing was independently performed according to the MM criteria (10) and an imaging algorithm based on lesion types and morphology (13).

### 2.3 WES, mutation calling, and RNA-Seq data processing

DNA from tissue samples and white blood cells (normal control) were isolated using the QIAamp DNA FFPE Tissue Kit and DNeasy Blood kit (Qiagen, Valencia, CA, USA), separately. DNA was quantified using the Qubit 2.0 Fluorometer (Life Technologies, Carlsbad, CA, USA) followed by qualification using Bioanalyzer 2100 (Agilent Technologies, Santa Clara, CA, USA). Genomic DNA was sheared into approximately 250-bp fragments by M220 Focused-ultrasonicator (Covaris, Woburn, MA, USA). A whole-genome library was prepared using the KAPA Hyper Prep Kit (KAPA Biosystems, Inc., Woburn, MA, USA). Whole-exome capture was performed using the xGen™ Exome Hybridization Panel (Integrated DNA Technologies, Coralville, IA, United States) according to the manufacturer’s protocol. Captured libraries were amplified using Illumina p5 and p7 primers in KAPA HiFi HotStart ReadyMix (KAPA Biosystems) and purified using Agencourt AMPure XP beads. Enriched libraries were sequenced using the Illumina HiSeq 4000 platform as paired-end 150-bp reads according to the manufacturer’s instructions. The targeted sequencing depth was 300×.

Mutation calling was performed as previously described (19–21). In brief, Trimmomatic (v0.36) was used for FASTQ file quality control (22). High-quality reads were aligned to the reference human genome (hg19, GRCh37) through Burrows-Wheeler aligner v0.7.12 (23). Duplication was removed with Picard, and local realignment around indels and base quality score recalibration was performed with the Genome Analysis Toolkit (GATK v3.2) (24). Somatic single-nucleotide variants (SNVs) and short insertions/deletions (indels) were identified by VarScan2 (25). SNVs and indels were retained according to the following criteria: 1) ≥4 variant supporting reads and ≥2% variant allele frequency supporting the variant, 2) filtered if present in >1% population frequency in the 1000g or ExAC database, and 3) filtered through an internally collected list of recurrent sequencing errors on the same sequencing platform. The final list of mutations was annotated using vcf2maf.

Total RNA from FFPE samples was extracted using Rneasy FFPE kit (QIAGEN, Valencia, CA, USA), and ribosomal RNA was depleted using Rnase H followed by library preparation using KAPA Stranded RNA-Seq Kit with RiboErase (KAPA Biosystems). Library concentration and quality were assessed using the KAPA Library Quantification Kit (KAPA Biosystems) and the Agilent High Sensitivity DNA kit on Bioanalyzer 2100 (Agilent Technologies), respectively. The library was then sequenced on Illumina HiSeq NGS platforms (Illumina, San Diego, CA, USA). Base calling was performed on bcl2fastq v2.16.0.10 (Illumina) to generate sequence reads in FASTQ format. Quality control was performed with Trimmomatic (22). STAR (26) is used for transcriptome mapping followed by isoform and gene level quantification performed using RSEM (27). The complex cellular heterogeneity in tumor tissues inferring immune and stromal cells was analyzed using the xCell algorithm based on RNA-Seq data. The immune and stroma scores were the enrichment scores of all immune cell types and stromal cell types, respectively. The microenvironment score was generated as the sum of all 64 immune and stromal cell types included by the xCell algorithm, which could be considered a novel measurement for tumor microenvironment abundance (28).

### 2.4 Tumor neoantigen prediction, mutational signature, phylogenetic tree, and gene similarity score

Human leukocyte antigen (HLA) typing of paired peripheral blood and tumor samples was performed using OptiType (29). All non-synonymous mutations and indels were translated into 21-mer peptide sequences using in-house software centered on mutated amino acids, followed by creating a 9- to 11-mer peptide via a sliding window approach to MHC class I binding affinity prediction. NeoPredPipe was used to predict the binding strength of mutated peptides to patient-specific HLA alleles (30). A peptide with predicted binding affinity to any HLA allele with IC50 < 500 nM (strong binder) was selected. Several selected peptides generated from the same mutation were counted as one neoantigen. Phylogenetic trees were built using non-synonymous somatic mutations (31, 32). Gene similarity score was calculated to present the relative probabilities of two samples having the same clonal origin (33).

### 2.5 Statistical analysis

Principal component analysis (PCA) for RNA-Seq data was performed on Fragments per Kilobase Million data using the *prcomp*, *complete*, and *hclust* packages in R software (version 4.1.2). The heatmap of stroma score, immune score, and microenvironment score was obtained by GraphPad Prism (version 8.3.1).

## 3 Results

### 3.1 Patient overview and serial sample collection

**Figure 1:**
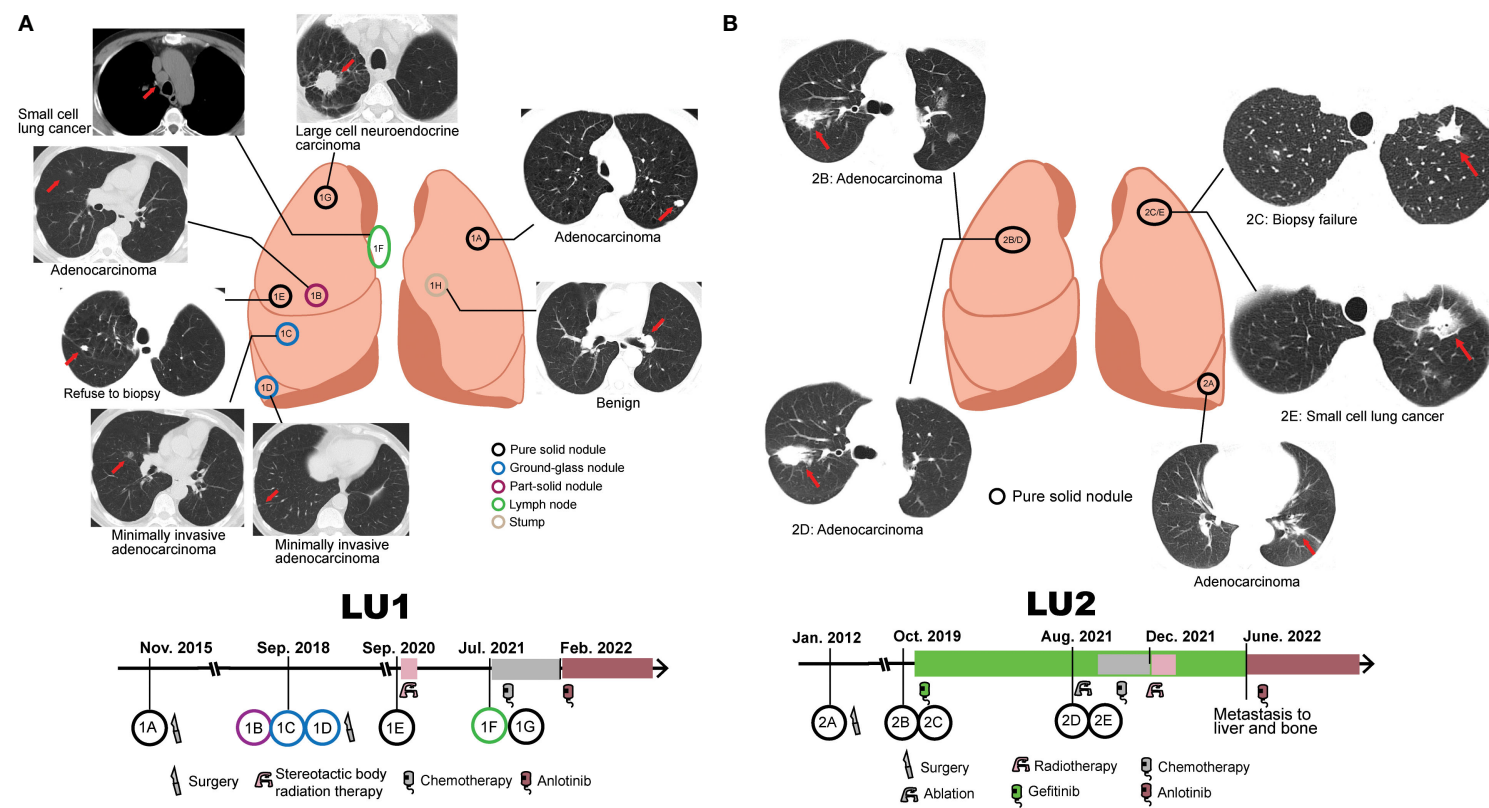
Collection of serial samples and treatment history. **(A)** Patient LU1’s six tumor tissue samples and one benign stump sample were collected. The treatment history of patient LU1 is shown below the position of tumor lesions. **(B)** Patient LU2’s four tumor tissue samples were collected. The treatment history of patient LU2 is shown below the position of tumor lesions. Red arrows indicate tumor lesions.

LU1, a 63-year-old male lung cancer patient, was a 30-pack-year smoker with a medical history of cerebral infarction in the right basal ganglia. In 2015, he presented with complaints of a non-productive cough. Chest CT demonstrated a solitary pulmonary nodule in the left upper lobe, which was defined as a well-differentiated adenocarcinoma with acinar predominant after left upper lobe lobectomy (LU1A, **Figure 1A**). LU1 presented with synchronous multiple ground-glass nodules and part-solid nodules in the right upper/lower lobe through CT scans in 2018. Histological assessment after partial lobectomy revealed one moderately differentiated adenocarcinoma with acinar predominant (LU1B) and two separate foci of MIA (LU1C and LU1D). In 2020, the patient refused a lung biopsy for another right upper lobe solitary nodule (LU1E) and underwent stereotactic body radiation therapy. Serial CT scans showed progression of one right upper lobe mass enlarging from 11 mm to 41 mm with mediastinal lymphadenopathy. The histological results of endobronchial ultrasound-guided transbronchial needle aspiration and CT-guided biopsy revealed an SCLC at the lymph node (LU1F), an LCNEC in the right upper lobe (LU1G), and a left lobe stump without detectable cancer cells (LU1H). Afterward, the patient was treated with first-line chemotherapy [cisplatin and etoposide, best response: stable disease (SD)], followed by second-line chemotherapy (irinotecan, best response: SD) and third-line anlotinib treatment (best response: SD).

LU2, a 72-year-old woman without a smoking history, was accidentally found to have one 1.6-cm well-defined nodule in the left lower lobe in 2012. The patient received a left lower lobectomy, and the pathology disclosed acinar predominant adenocarcinoma with visceral pleural elastic layer infiltration (LU2A, **Figure 1B**). In 2019, a CT scan demonstrated synchronous bilateral ground-glass opacity and solid nodules, including an acinar predominant adenocarcinoma (LU2B) and a failed biopsy of the left upper lobe nodule (LU2C). The patient was treated with gefitinib due to detectable *EGFR^L747_T751del^* mutation in LU2B and refusal to surgical intervention, with the best response of SD. In 2021, the patient underwent endobronchial ultrasound-guided transbronchial lung biopsy and electromagnetic navigation bronchoscope biopsy at the same locations as LU2B and LU2C, respectively. An acinar predominant adenocarcinoma nodule (LU2D) and an SCLC nodule (LU2E) were identified. The patient received ablation, cisplatin, and etoposide plus gefitinib (best response: SD) and radiotherapy (best response: SD) sequentially. Due to the metastasis to the liver and bone in June 2022, she had been treated with anlotinib by the end of the follow-up.

### 3.2 Lineage tracing by the multi-omics analysis method

**Figure 2:**
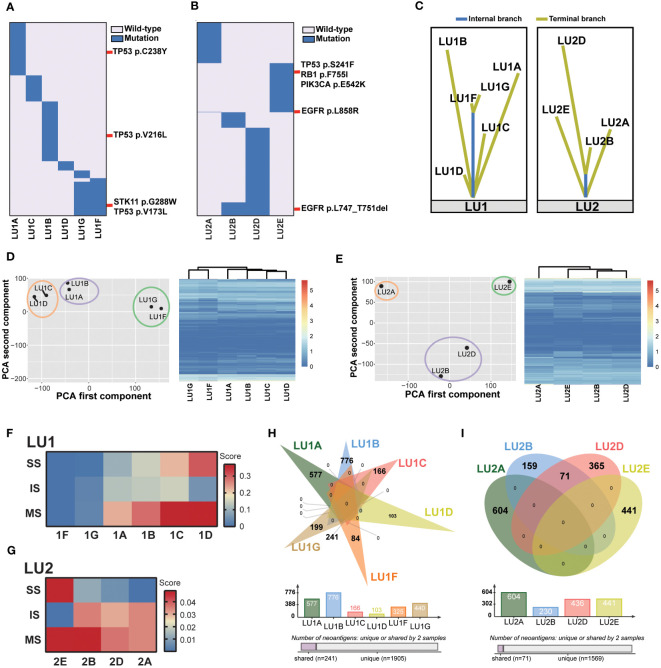
Lineage tracing by the multi-omics analysis method. **(A)** The mutational landscape of patient LU1’s six tumor tissue samples profiled by whole-exome sequencing. No somatic mutations were detected in the benign stump sample. Potential driver mutations, such as *TP53^V173L^*, *TP53^V216L^*, *TP53^C238Y^*, and *STK11^G288W^*, are marked. **(B)** The mutational landscape of patient LU2’s four tumor tissue samples profiled by whole-exome sequencing. Driver mutations, such as *EGFR^L858R^* and *EGFR^L747_T751del^*, are marked. **(C)** The lineage of two patients’ samples traced by the phylogenetic trees. **(D, E)** Patients LU1’s and LU2’s RNA expression features by PCAs and unsupervised cluster analyses. **(F)** The tumor immune microenvironment characterized by RNA-Seq data, including stroma scores (SS), immune scores (IS), and microenvironment scores (MS) of patient LU1’s samples generated by the xCell algorithm. Both LU1F and LU1G had relatively low scores when compared to other four samples. **(G)** The tumor immune microenvironment characterized by RNA-Seq data. LU2B and LU2D displayed similar scores, suggesting potentially similar tumor microenvironment abundance. **(H, I)** Predicted tumor neoantigens were shared between LU1F and LU1G, as well as LU2B and LU2D, whereas no common predicted tumor neoantigens were observed in other samples.

As two specimens were not eligible for the multi-omics analysis method (LU1E and LU2C: refuse/failure to tumor tissue biopsy), the lineage of the remaining 11 tumor tissue biopsies was traced. WES data revealed no shared mutations in samples LU1B, LU1C, and LU1D, which were multiple synchronous tumors observed in 2018, suggesting a high probability of MPLC (**Figure 2A**). When compared to sample LU1A, these three samples also did not share any mutations. In comparison with LU1F, LU1G had considerable shared mutations (gene similarity score: 75.1%), which suggests a potential for IPM. Of note, no somatic mutations were detected in LU1H, which was also pathologically considered to be benign. The WES data of LU2 also aided in lineage tracing (**Figure 2B**). Considering the location of biopsies as well as the multiple shared mutations between LU2B and LU2D (gene similarity score: 12.9%), especially the same driver mutation of *EGFR^L747_T751del^*, we supposed that LU2D was a progression lesion derived from LU2B; however, neither LU2B nor LU2D might be the recurrence/metastasis of LU2A owing to different *EGFR* drivers (*EGFR^L747_T751del^ vs. EGFR^L858R^*) as well as no detectable shared mutations. Although both LU2A and LU2E were identified with the same *EGFR^L858R^* driver mutation, other shared mutations were rarely observed (gene similarity score: 0.4%), suggesting a low possibility of LU2E being a recurrence/metastasis of LU2A. However, due to the failure of the LU2C biopsy, we were not able to directly determine the lineage of LU2C and LU2E. Considering gefitinib treatment after the diagnosis of LU2B and LU2C, as well as detectable *EGFR*, *TP53*, and *RB1* mutations in LU2E, we supposed that LU2E might result from the histological transformation related to gefitinib resistance. Moreover, the lineage of two patients’ samples traced by mutational landscape was confirmed by the phylogenetic trees (**Figure 2C**).

Next, the lineage tracing based on WES data was double-checked using RNA-Seq data by PCA and unsupervised cluster analyses. The position of each tumor sample in a 2D coordinate system depended on the first and second components using PCAs. For patient LU1’s six tumor samples, two adenocarcinoma samples (LU1A and LU1B) clustered together; LU1C and LU1D, which were two MIA samples, were close to each other (**Figure 2D**). These results demonstrated that lung cancer samples of the same histological type showed similar RNA expression. Notably, LU1F and LU1G also clustered together, being significantly distant to the other four adenocarcinoma or MIA samples even though LU1F and LU1G were SCLC and LCNEC, respectively, suggesting the possibility that LU1F had the same lineage as LU1G and the likelihood of LCNEC transforming into SCLC during metastatic invasion. The clustering analyses also revealed that LU1F and LU1G had similar RNA expression patterns (**Figure 2D**). Patients LU2, LU2B, and LU2D presented similar RNA expression features, with relatively close locations when compared to LU2A and LU2E (**Figure 2E**), which was consistent with the clustering analyses (**Figure 2E**). The results of xCell demonstrated relatively low scores of immune-related cells in multiple samples of LU1, such as CD8^+^ T cells and NK cells (**Supplementary Figure 1**). Of note, samples LU1B, LU1C, and LU1D appeared to have higher scores of B cells, NK T cells, endothelial cells, and hematopoietic stem cells than LU1F and LU1G. By contrast, LU1F and LU1G were more likely to have relatively high scores of CD4^+^ Th1 and Th2 T cells (**Supplementary Figure 1**). Additionally, the xCell algorithm revealed that both LU1F and LU1G had relatively low stroma scores, immune scores, and microenvironment scores in comparison with patient LU1’s other four tissue samples (**Figure 2F**). However, LU1B, LU1C, and LU1D showed distinctive stroma scores, immune scores, and microenvironment scores, suggesting that these three samples might be MPLC (**Figure 2F**). For patient LU2’s samples, LU2A, LU2B, and LU2D appeared to have similar tumor microenvironment landscape, except for the higher hematopoietic stem cell score in LU2D than in LU2A and LU2B (**Supplementary Figure 2**). The stroma, immune, and microenvironment scores of four samples were low, ranging from 0 to 0.05, whereas LU2E appeared to have a higher stroma score and lower immune score when compared to the other three samples even though the overall microenvironment score of LU2E was close to LU2B (**Figure 2G**). LU2B and LU2D displayed similar stroma, immune, and microenvironment scores, suggesting potentially similar tumor microenvironment abundance. Therefore, RNA-Seq results revealed that LU1F and LU1G could possibly be identified as IPM. However, due to the RNA expression similarity in the same histology subtype and patient LU2’s samples, the lineage of LU1C and LU1D as well as that of LU2A, LU2B, and LU2D might not be identified clearly using RNA-Seq data alone.

As the RNA-Seq data indicated a potentially similar tumor microenvironment between LU1F and LU1G, as well as LU2A, LU2B, and LU2D, the tumor neoantigens were further compared. The same neoantigens were exclusively observed between LU1F and LU1G in patient LU1’s samples, with 74.2% of neoantigens of LU1F and 54.7% of neoantigens of LU1G being shared, which supported that these two samples were IPM (**Figure 2H**). By contrast, all LU1A, LU1B, LU1C, and LU1D were predicted with unique neoantigens, suggesting that LU1B, LU1C, and LU1D might be determined as MPLC without a high possibility of relapse of LU1A. Similarly, 71 shared neoantigens were observed between LU2B (30.9%) and LU2D (16.3%), which demonstrated the potential that LU2D was a relapse of LU2B (**Figure 2I**). Considering no neoantigens were shared between LU2D and LU2E, there was a low possibility that LU2E was the intrapulmonary metastasis of LU2D.

### 3.3 Validation by clinicopathologic and molecular criteria

**Figure 3:**
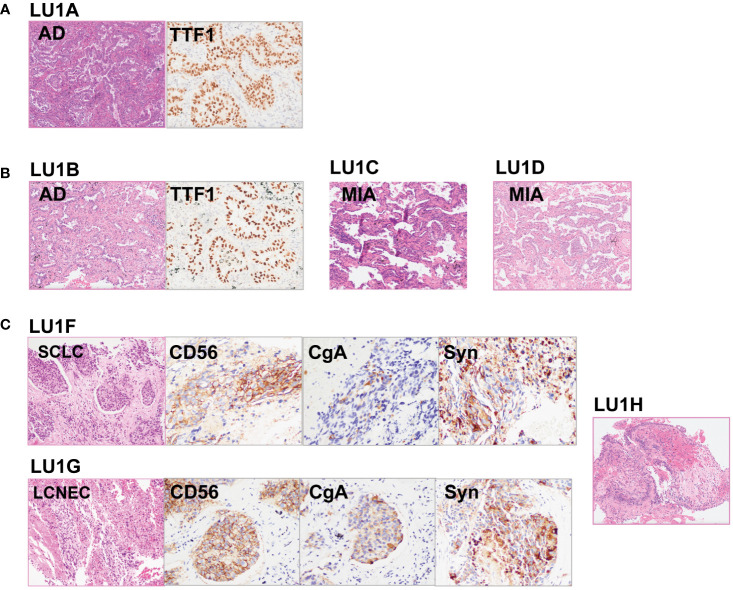
Histological reviews for patient LU1’s biopsies. **(A)** Histological reviews for sample LU1A collected in November 2015. **(B)** Histological reviews for samples LU1B, LU1C, and LU1D collected in September 2018. **(C)** Histological reviews for samples LU1F, LU1G, and LU1H collected in July 2021.

**Figure 4:**
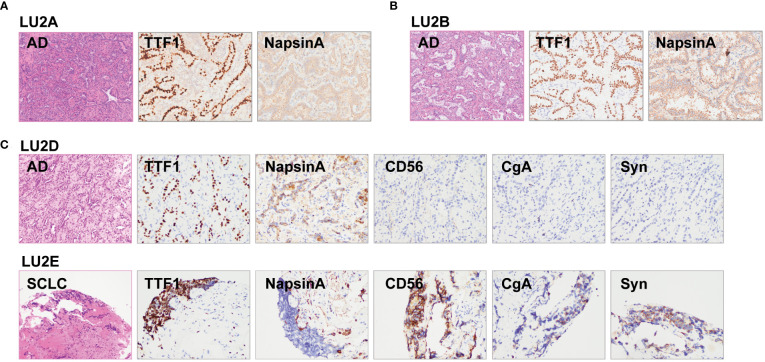
Histological reviews for patient LU2’s biopsies. **(A)** Histological reviews for sample LU2A collected in January 2012. **(B)** Histological reviews for sample LU2B collected in October 2019. **(C)** Histological reviews for samples LU2D and LU2E collected in August 2021.

The lineage determined using the multi-omics analysis method is summarized in **Table 1**. To validate the accuracy of this novel method, the findings were further confirmed through additional validation strategies, including pathology, MM criteria, and imaging algorithms. Histological review (**Figure 3**) and MM criteria reached an agreement that tumors of LU1 were characterized as primaries due to different histological types or physical separation, except for LU1B, whose lineage was indeterminate by pathologists’ histological review (**Table 1**). Immunohistochemical staining showed that LU1F and LU1G were both positive for synaptophysin, chromogranin A, and CD56 (**Figure 3C**). The imaging algorithm suggested that LU1B–D were MPLC and that LU1F was a metastasis of synchronous LU1G. The multi-omics analysis method also identified LU1B–D as MPLC without an obvious relationship with LU1A, and LU1F and LU1G were determined as IPM. For patient LU2 (**Figure 4**), pathologic interpretation confirmed LU2A and LU2E as primary, whereas the lineage of LU2B and LU2D remained indeterminate when compared to that of sample LU2A (**Table 1**). The histological review identified LU2E as an SCLC sample, showing positive staining for chromogranin A, synaptophysin, and CD56, but negative for NapsinA, whereas LU2D was identified as an acinar predominant adenocarcinoma, with negative staining for chromogranin A and synaptophysin (**Figure 4C**). In contrast, the MM criteria identified LU2D as the progression of LU2B, and LU2E as the progression of LU2C. The imaging algorithm demonstrated a diagnosis of MPLC for LU2B and LU2C and progression for LU2D and LU2E from their original lesions. The multi-omics analysis method revealed that LU2D was a progression lesion derived from LU2B, whereas it suggested a potential histological transformation from LU2C to LU2E.

**Table 1:** Lineage tracing by the multi-omics analysis method and traditional approaches.

Patient	Time point	Sample	Lineage tracing approaches			
			Pathology	MM criteria	Imaging	Multi-omics
LU1	Nov 2015	LU1A	Primary	Primary	Primary	Primary
	Sep 2018	LU1B	Indeterminate	MPLC	MPLC	MPLC
		LU1C	MPLC			
		LU1D				
	Sep 2020	LU1E	NA	NA	Primary	NA
	Jul 2021	LU1F	MPLC	MPLC	IPM	IPM
		LU1G				
		LU1H	Benign	NA	NA	Benign
LU2	Jan 2012	LU2A	Primary	Primary	Primary	Primary
	Oct 2019	LU2B	Indeterminate	Primary	MPLC	Primary
		LU2C	NA	NA		NA
	Aug 2021	LU2D	Indeterminate	Progression of 2B	Progression of 2B	Progression of 2B
		LU2E	Primary	Progression of 2C	Progression of 2C	2C SCLC transformation

MM, Martini and Melamed; MPLC, multiple primary lung cancer; NA, not applicable; SCLC, small cell lung cancer.

## 4 Discussion

In this study, we developed a multi-omics analysis method integrating WES, RNA-Seq, and tumor neoantigen data to aid in lineage tracing for multiple lung cancer by spatiotemporal heterogeneity. This molecular feature-based approach achieved good performance in serial tissue samples of two patients with complicated surgical and systemic treatment histories. Compared with previous lineage tracing approaches based on histological review, MM criteria, and imaging characteristics, the novel multi-omics method was able to define MPLC and IPM with high agreement and overcome the shortage of inter-observer variability.

Neither histological nor molecular features alone were perfect classifiers for lineage tracing resulting in discordance of lineage (4), and the combination of histological and molecular features remained insufficient to predict lineage or determine the best course of treatment, suggesting a need for a more objective and comprehensive approach. The genomic profile-based algorithm might be a promising method to define lineage in multiple lung cancer, as two-thirds of patients would perform molecular analysis to assess the genetic agreements of different lesions in a recent global survey on the management of multiple lung cancer (34). WES demonstrated comprehensive detection of shared and unique mutations of multiple lung cancer. Our combined method, which delineated spatiotemporal heterogeneity of series tumor tissue samples through genomic, transcriptomic, and immune-related tumor neoantigen, was an effective alternative in improving the accuracy of tracing lineage and tracking the evolutionary process. Similar to the findings of our research, a previous study where both genomic and transcriptomic features were used to generate a combined classifier achieved a better performance than using one feature alone (area under the curve for the combined classifier, 0.79; WES alone, 0.69; RNA-Seq alone, 0.73) (35). Immune scores, summarizing the density, type, and proportion of tumor-infiltrating lymphocytes and reflecting the immune-related tumor microenvironment, also assisted in lineage calling. These results highlighted the clinical significance of the dynamic multi-omics landscape in phenotype characterization, tumor staging, and governing. Aligned with the similarities and differences revealed by immune scores, the same tumor neoantigens predicted by somatic mutations and indels were shared between samples with similar immune-related microenvironments.

As an auxiliary tool to distinguish MPLC from IPM, comprehensive histological subtyping and imaging characteristics might reflect molecular characteristics instead of accurately predicting the underlying genetic similarities and differences between MPLC and IPM. The International Association for the Study of Lung Cancer proposal mentioned that paired tumor samples in which at least one lesion presented with ground-glass opacity nodular features reflected multifocal lung adenocarcinoma with ground glass/lepidic features (13). There was also a significant association between *KRAS* mutations and MPLC development in patients exposed to tobacco (36). The American Joint Committee on Cancer (AJCC) staging demonstrated the positive status of lymph nodes, which was associated with overall survival (*p* = 0.0001), as a symbol of IPM rather than MPLC (4, 9). However, the positivity of the lymph node that was not able to predict molecular similarity might lead to lineage misclassification. For instance, when detected with LU1G and LU1F, which was a potential metastasis of LU1G, patient LU1 might receive surgery or stereotactic body radiation therapy if LU1G and LU1F were histologically considered as MPLC without the aid of the multi-omics analysis method. Although chemotherapy might not be applied alternatively according to the molecular feature-based algorithm, treatment was possibly changed toward limited parenchymal resections or stereotactic body radiation therapy for MPLC and solitary IPM in selected cases (37).

The discordance of lineage tracing for certain samples between pathology/MM criteria and the multi-omics analysis method resulted from different histological types of serial samples. For example, LU1F and LU1G, which were SCLC and LCNEC, respectively, were identified as primaries owing to completely different histological types. However, the genomic and transcriptomic landscapes suggested the two samples’ relationship behind different histological types. This result reminded us that spatiotemporal heterogeneity and the evolutionary relationship of multiple lung cancer could be accurately unveiled by genomic and transcriptomic features. The discordance of lineage determination for LU2E might also be rationalized by different histological types, in addition to the lack of LU2C biopsy. Integrating the *EGFR* driver mutation, the contaminant *TP53* and *RB1* mutations (38, 39), and the use of gefitinib, the multi-omics analysis method suggested that LU2E, a SCLC sample, was likely to result from the histological transformation rather than the development of a primary lesion, even though there was no molecular feature information of LU2C. Due to the failure of tissue biopsy of LU2C, neither pathology nor MM criteria were able to reveal the lineage between LU2C and LU2E; however, the imaging algorithm, which did not rely on successful biopsy, could also flag the potential relationship between these two samples.

In our study, MPLC exhibited strong heterogeneity of molecular characteristics, such as rarely shared mutations except for common lung cancer driver mutations. Our findings were consistent with a previous study indicating that the genomic similarity among one lung cancer patient’s independent primaries was not relatively high in comparison with a tumor randomly selected from The Cancer Genome Atlas (TCGA) lung adenocarcinoma database (40). Moreover, our RNA-Seq results revealed that MPLC with various histological types had different RNA expression patterns, consistent with the finding that RNA expression could help differentiate lung cancer histology (41, 42). Interestingly, two patients’ adenocarcinoma samples with few shared mutations had similar RNA expression patterns, and similar findings were observed in patient LU1’s two independent MIA primary samples. Owing to the limited sample size, this finding requires further investigation in a large study cohort including more independent primary lesion samples of different histological types.

Heterogeneous tumor cells with different gene expression, tumor–host interactions, and potential biological behaviors were influenced by tumor cell-intrinsic genetic and epigenetic determinants (6). Currently, tumor heterogeneity is generally interpreted using the trunk-branch model. Trunk gene mutations drove tumor growth in subcloning and tumor regions, while the branch ones induced tumor heterogeneity in primaries and metastases (43). An analysis of the first 100 patients enrolled in TRACERx based on WES analysis revealed pervasive genomic heterogeneity (44). Given the pervasiveness and importance of tumor heterogeneity in the relapse patterns and prognosis, WES and RNA-Seq analyses were proposed to reveal significant genomic–transcriptomic heterogeneity and complex evolutionary process in identical tumors with different sampling time (LU2B/D) or undergoing cancer treatment (LU2C/E) with or without the retaining of core genome.

This study has limitations. Only two patients were enrolled in this study, and there was a lack of key tumor tissue samples. For example, sample LU2C, which could provide critical information about the lineage determination, was not collected unsuccessfully, leading to the failure to directly define the association between LU2C and LU2E. Although only two patients were included, their multiple samples spanned across different time points, and these two patients underwent various treatment regimens. As a preliminary investigation, this study demonstrated the feasibility of utilizing multidimensional molecular features; however, future studies with larger sample sizes are essential to validate the accuracy of lineage calling by the multi-omics analysis method, including sensitivity and specificity compared to conventional histological assessment and imaging methods. Also, sample LU1F was obtained through endobronchial ultrasound-guided transbronchial needle aspiration from a lymph node, which might lead to inaccurate lineage tracing due to the small volume. Another limitation is that some tumor tissue samples were collected several years ago, possibly resulting in the accuracy issue of RNA-Seq results. Further well-designed prospective studies are warranted to confirm the role of RNA-Seq in helping lineage tracing. Additionally, the impact of the multi-omics analysis method on treatment strategy development remained incomprehensively investigated in this retrospective study, even though the multi-omics analysis method was able to predict the lineage more reasonably than the traditional pathology approach and the MM criteria. Thus, further prospective studies are required to assess its benefits in treatment plan modification and prognosis by correcting potential lineage determination mistakes.

In summary, the multi-omics analysis method revealing spatiotemporal heterogeneity by genomic, transcriptomic, and immune-related characteristics may be an alternative approach to accurate lineage tracing for multiple lung cancer, with a promise in clinical application for disease management assistance and prognosis improvement, while further validation in large cohorts is warranted.

## Data availability statement

The original contributions presented in the study are publicly available. This data can be found here: https://ngdc.cncb.ac.cn/gsa-human/browse/HRA004862.

## Ethics statement

The studies involving humans were approved by the Ethics Committee of Zhongshan Hospital Fudan University (Approval Number: B2017-142R). The studies were conducted in accordance with the local legislation and institutional requirements. The participants provided their written informed consent to participate in this study. Written informed consent was obtained from the individual(s) for the publication of any potentially identifiable images or data included in this article.

## Author contributions

JH designed the study. YS, JZ, YZ, XX, and DZ were responsible for patient recruitment and clinical data collection. YJ, MZ, and YW were responsible for histological reviews. YS, JZ, and JH performed lineage tracing by clinicopathologic reviews and the imaging-based algorithm. JP, HB, and QO were in charge of sequencing data processing. YS, JZ, JP, HB, XZ, and JH analyzed the data and interpreted the results. YS and XZ wrote the first draft of the manuscript. All authors contributed to the article and approved the submitted version.

## Supplementary material

Supplementary Figure 1 RNA-Seq results for patient LU1’s biopsies by the xCell algorithm. Relatively low scores of immune-related cells were observed in multiple samples of LU1, such as CD8+ T-cells and NK cells. Sample LU1B, LU1C, and LU1D appeared to have higher scores of B cells, NK T-cells, endothelial cells, and hematopoietic stem cells than LU1F and LU1G that were more likely to have relatively high scores of CD4+ Th1 and Th2 T cells.

Supplementary Figure 2 RNA-Seq results for patient LU2’s biopsies by the xCell algorithm. LU2A, LU2B and LU2D appeared to have similar tumor microenvironment landscape, but a higher hematopoietic stem cell score was observed in LU2D than in LU2A and LU2B.&
