# Supplementary figures and images for: Lineage tracing for multiple lung cancer by spatiotemporal heterogeneity using a multi-omics analysis method integrating genomic, transcriptomic, and immune-related features
Source: Front Oncol. 2023 Sep 14;13:1237308. doi: 10.3389/fonc.2023.1237308 (PMC10548834; doi:10.3389/fonc.2023.1237308)

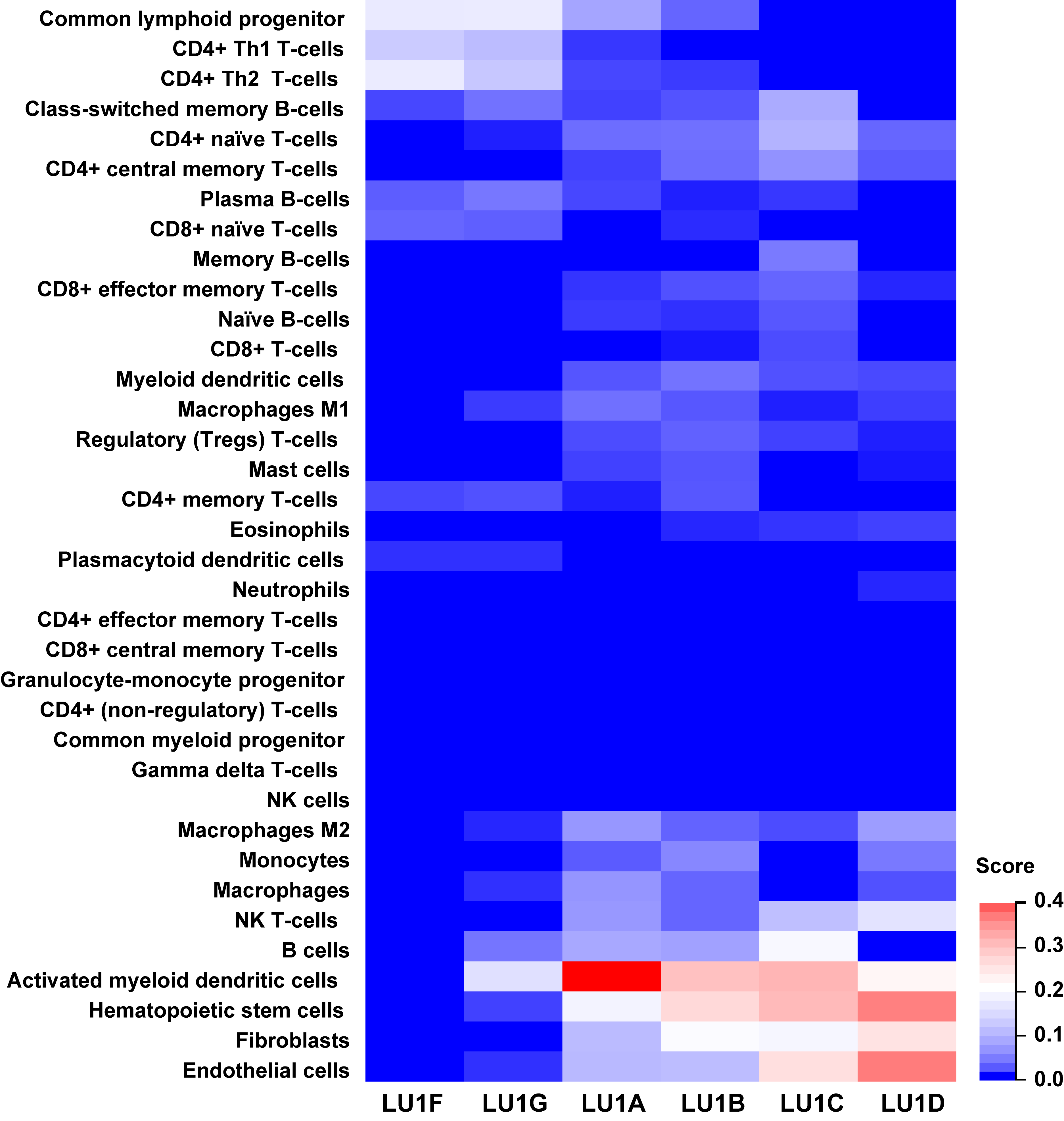

Supplement: Supplementary Figure 1 — RNA-Seq results for patient LU1’s biopsies by the xCell algorithm. Relatively low scores of immune-related cells were observed in multiple samples of LU1, such as CD8+ T-cells and NK cells. Sample LU1B, LU1C, and LU1D appeared to have higher scores of B cells, NK T-cells, endothelial cells, and hematopoietic stem cells than LU1F and LU1G that were more likely to have relatively high scores of CD4+ Th1 and Th2 T cells. [file Image_1.tif]

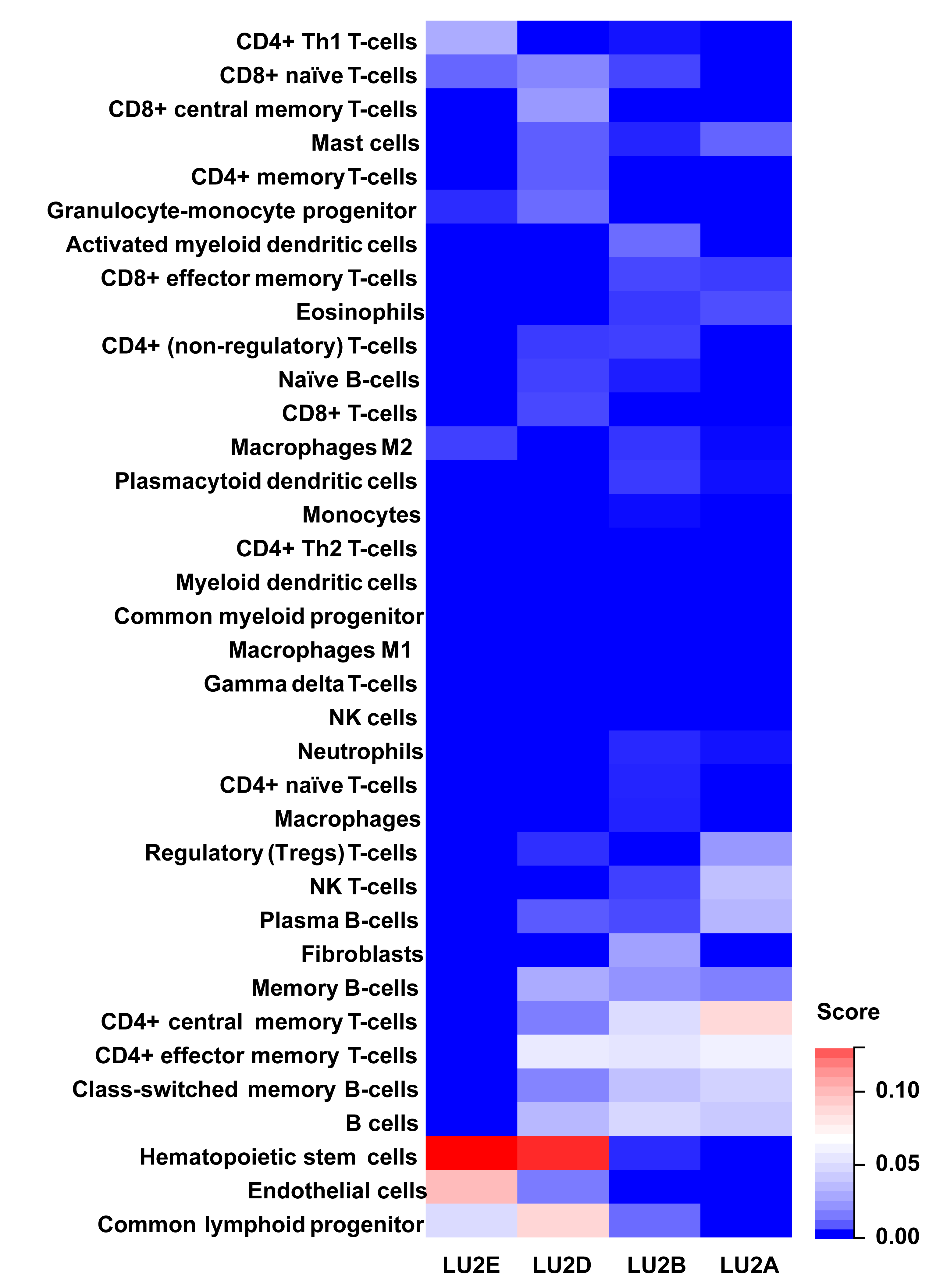

Supplement: Supplementary Figure 2 — RNA-Seq results for patient LU2’s biopsies by the xCell algorithm. LU2A, LU2B and LU2D appeared to have similar tumor microenvironment landscape, but a higher hematopoietic stem cell score was observed in LU2D than in LU2A and LU2B.& [file Image_2.tif]
